# Supplementary material for: Vimentin binds to G-quadruplex repeats found at telomeres and gene promoters
Source: Nucleic Acids Res. 2022 Jan 31;50(3):1370–81. doi: 10.1093/nar/gkab1274 (PMC8860586; doi:10.1093/nar/gkab1274)
Supplement: gkab1274_Supplemental_Files [file gkab1274_supplemental_files.zip › SUPPLEMENTARY MATERIAL EXCEL FILE LEGENDS.docx]

SUPPLEMENTARY MATERIAL EXCEL FILE

**LEGENDS**

**Table S1. Proteins from pull-down assays identified by LC-MSE:** Protein Score obtained in the Mascot search and the significant matched peptides for the proteins identified in gel bands V, S2 and S1 (Table 1, main text). Reported in **Sheet LC-MSE**

**Table S2. LC-MS analyses of the digests of the gel bands derived from limited proteolysis:** with reference to Figure 4 data for bands 0, 1 2 3 4 are reported in **SHEETS Band 0_VIM 0min; Band 3_VIM 1min; Band 4_VIM 1min; Band 1_VIM+DNA 1min; Band 2_VIM+DNA 1min; Band 3_VIM+DNA 1min; Band 4_VIM 1min; 4_VIM+DNA 5min**

**SHEET double_triple_G4_PQS descr.:** List of sequences containing either a double or triple G4 repeat. Presence of a double and/or a triple G4 repeat is checked as an 'x' in column E and F.

**SHEET double_triple_G4_PQS list:** Ensembl ID accession numbers of sequences containing either a double or triple G4 repeat.

**SHEET GC_rich_BKG list:** Ensembl ID accession numbers of sequences NOT containing a double or a triple G4 repeat and having a GC content higher than 60% in the 100bp upstream the TSS site.

**SHEET Biological Process:** Gene Ontology (GO) terms from Biological Process (BP).

Enriched GO terms are reported according to DAVID enrichment analysis. Double-triple G4 repeats are compared to the background list of human genes with a high GC content but not containing double-triple G4 repeats.

The Pareto chart reports GO FAT BP enriched analysis from DAVID (FAT: filters out very broad GO terms based on a measured specificity of each term) at a Bonferroni-adjusted p-value < 0.05.

379 genes from double-triple PQSs do not contain GO annotations in BP and are consequently not considered in the analysis.

**SHEET Molecular Function:** Gene Ontology (GO) terms from Molecular Function (MF).

Enriched GO terms are reported according to DAVID enrichment analysis. Double-triple G4 repeats are compared to the background list of human genes with a high GC content but not containing double-triple G4 repeats.

The Pareto chart reports GO FAT MF enriched analysis from DAVID (FAT: filters out very broad GO terms based on a measured specificity of each term) at a Bonferroni-adjusted p-value < 0.05.

546 genes from double-triple G4 repeats do not contain GO annotations in MF and are consequently not considered in the analysis.

**SHEET Cellular Component:** Gene Ontology (GO) terms from Cellular Component (CC).

Enriched GO terms are reported according to DAVID enrichment analysis. Double-triple G4 repeats are compared to the background list of human genes with a high GC content but not containing double-triple G4 repeats.

The Pareto chart reports GO FAT CC enriched analysis from DAVID (FAT: filters out very broad GO terms based on a measured specificity of each term) at a Bonferroni-adjusted p-value < 0.05.

708 genes from double-triple G4 repeats do not contain GO annotations in CC and are consequently not considered in the analysis.

**SHEET Functional Clustering:** Functional Annotation Clustering analysis.

Enriched GO terms are reported according to DAVID functional annotation clustering analysis. Double-triple G4 repeats are compared to the background list of human genes with a high GC content but not containing double-triple G4 repeats.

The Pareto chart reports the Functional Annotation Clustering of DAVID performed on GO_TERM_BP_FAT, GO_TERM_CC_FAT, GO_TERM_MF_FAT, keywords, and KEGG pathways at a Bonferroni-adjusted p-value < 0.05.

**SHEET GC content:** Quantification and frequency of Human genes containing vs nor containing double-triple G4 repeats within upstream 100bp of TSS.
