# Supplementary material for: Vimentin binds to G-quadruplex repeats found at telomeres and gene promoters
Source: Nucleic Acids Res. 2022 Jan 31;50(3):1370–81. doi: 10.1093/nar/gkab1274 (PMC8860586; doi:10.1093/nar/gkab1274)
Supplement: gkab1274_Supplemental_Files [file gkab1274_supplemental_files.zip › Supplementary Material_revised_clean.pdf]

SUPPLEMENTARY MATERIAL

Vimentin binds to G-quadruplex repeats found at telomeres and gene promoters

Silvia Ceschi[1], Michele Berselli[3], Marta Cozzaglio[1], Mery Giantin[4], Stefano Toppo[2,3,*], Barbara Spolaore[1,2] and Claudia Sissi[1,2,*]

[1] Department of Pharmaceutical and Pharmacological Sciences, University of Padova, Padova, 35131, Italy
[2] CRIBI Biotechnology Center (Centro di Ricerca Interdipartimentale per le Biotecnologie Innovative), University of Padova, Padova, 35131, Italy
[3]Department of Molecular Medicine, University of Padova, Padova, 35131, Italy
[4]Department of Comparative Biomedicine and Food Science, University of Padova, Legnaro, 35020, Italy

* To whom correspondence should be addressed. Tel: +390498275711; Fax: +390498275366; Email: claudia.sissi@unipd.it;
Correspondence may also be addressed to Stefano Toppo. Tel: +390498276958; Fax: +390498073310; Email: stefano.toppo@unipd.it;
Present Address: [Michele Berselli], Department of Biomedical Informatics, Harvard Medical School, Boston, MA, 02115, USA;

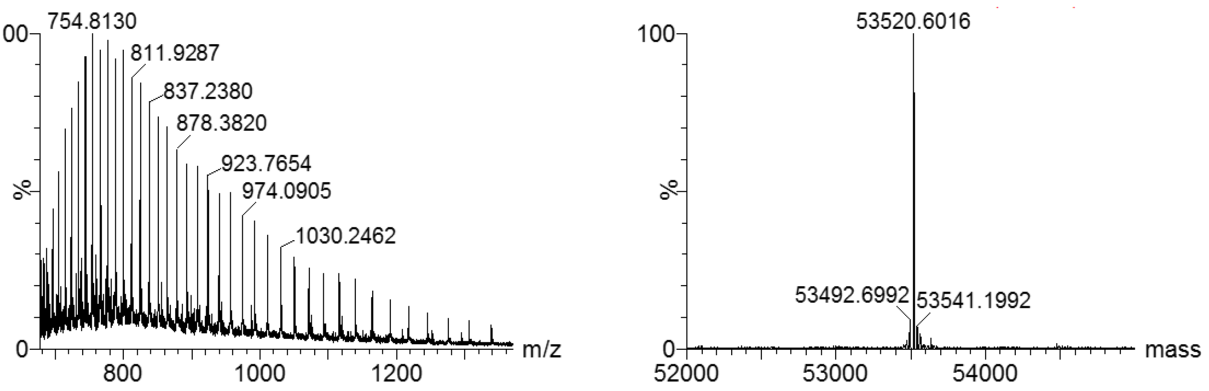

Figure S1. ESI-MS analysis of recombinant human Vimentin (calculated mass 53520.6 Da). The ESI mass spectrum before (left) and after deconvolution (right) is reported. The spectrum was acquired on the Xevo G2-S QTof operated in the ESI positive ion resolution mode.

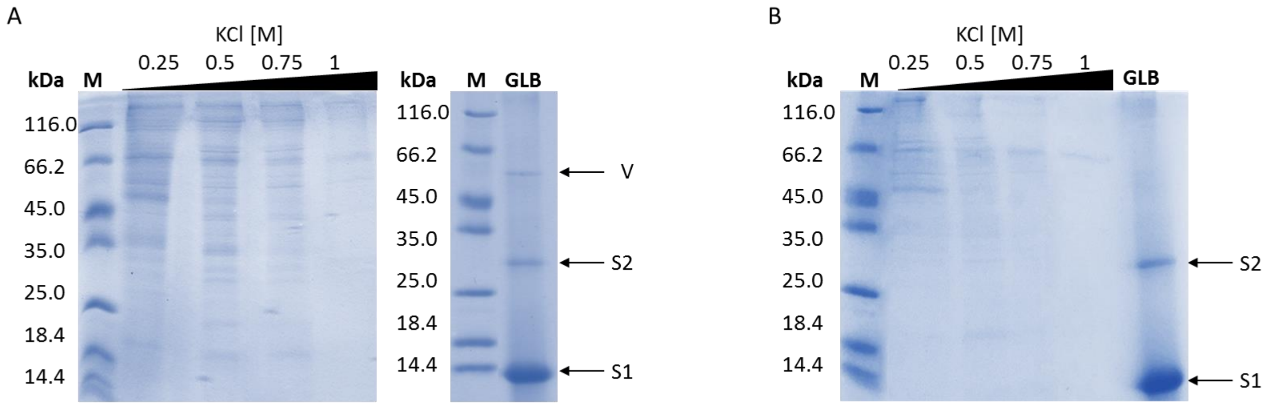

Figure S2. A) SDS-PAGE of the first fractions (on the left) and the last fraction (on the right) obtained from protein elution after pull-down assays performed with KIT2KIT* G-quadruplex. Bands corresponding to Streptavidin monomer (S1) and dimer (S2) and Vimentin (V) are highlighted. B) SDS-PAGE of the fractions obtained from protein elution after pull-down assays performed with KIT2KIT* duplex. Bands corresponding to Streptavidin monomer (S1) and dimer (S2) are highlighted.

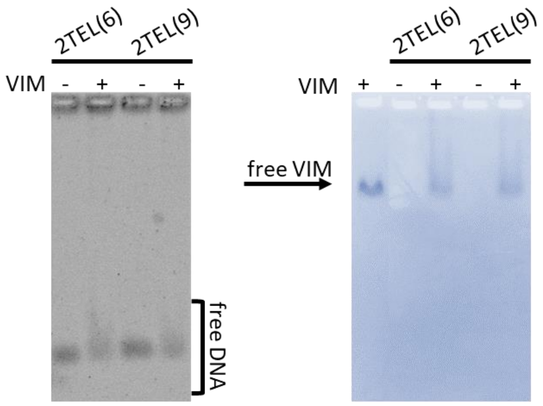

Figure S3. EMSA of 500 nM 2TEL(6) and 2TEL(9) in the presence (+) or absence (-) of 8 µM Vimentin in 5 mM Tris-HCl (pH 8.4), 150 mM KCl. Gels were stained with Sybr Green II (on the left) and with colloidal Coomassie Brilliant Blue G250 (on the right).

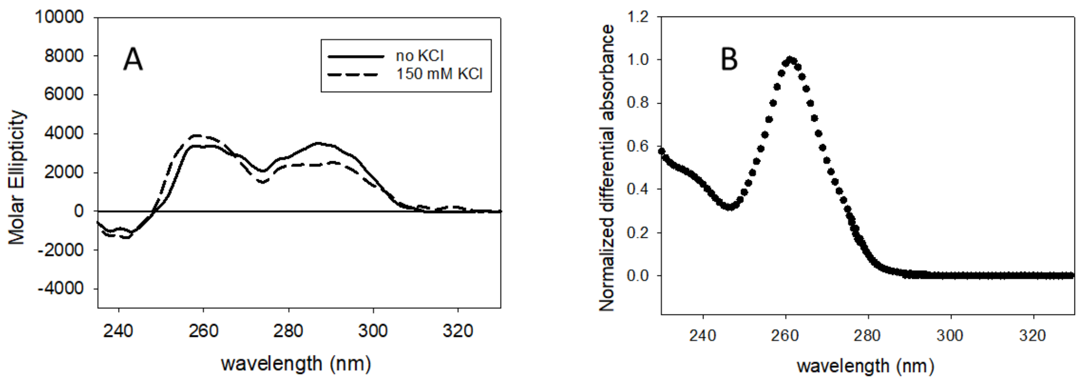

Figure S4. (A) CD spectra of 2 µM single-stranded G-rich 49-mers oligonucleotide (G-rich noG4) in 10 mM Tris-HCl (pH 7.4) before (solid line) and after addition of 150 mM KCl, at 25 °C (dashed line) and (B) thermal difference spectra (TDS) derived from subtraction of the oligonucleotide UV spectra acquired in 10 mM Tris-HCl, 150 mM KCl (pH 7.4) at 25 °C and 95 °C (1).

1. Mergny, JL., Li, J., Lacroix, L., Amrane, S., Chaires, J.B (2005) Thermal difference spectra: a specific signature for nucleic acid structures Nucleic Acids Res., 33, e138

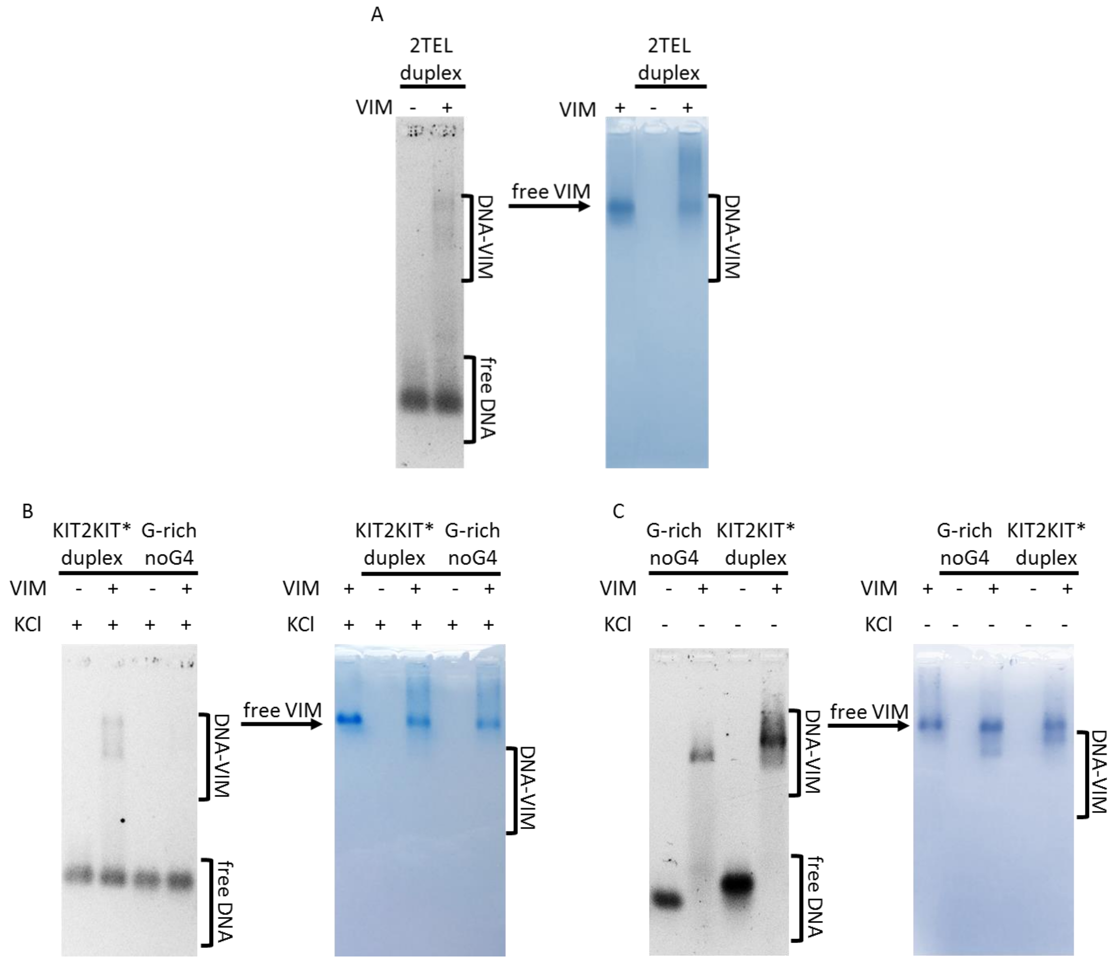

Figure S5. A) EMSA of 500 nM 2TEL paired with its complementary strand, with 8 µM Vimentin in 5 mM Tris-HCl (pH 8.4), 150 mM KCl, stained with Sybr Green II (on the left) and Coomassie Brilliant Blue G250 (on the right); EMSA of 500 nM KIT2KIT* paired with its complementary strand and 49-mers G-rich unfolded single-stranded oligonucleotide with 8 µM Vimentin in 5 mM Tris-HCl (pH 8.4) (B) in the presence and (C) in the absence of 150 mM KCl, stained with Sybr Green II (on the left) and Coomassie Brilliant Blue G250 (on the right).

STRSVSSSS YRRMFGGPGT ASRPSSSRSY VTTSTRTYSL GSALRPSTSR
60 70 80 90 100
SLYASSPGGV YATRSSAVRL RSSVPGVRLL QDSVDFSLAD AINTEFKNTR
110 120 130 140 150
TNEKVELQEL NDRFANYIDK VRFLEQQNKI LLAELEQLKG QGKSRLGDLY
160 170 180 190 200
EEEMRELRRQ VDQLTNDKAR VEVERDNLAE DIMRLREKLQ EEMLQREEAE
210 220 230 240 250
NTLQSFRQDV DNASLARLDL ERKVESLQEE IAFLKKLHEE EIQELQAQIQ
260 270 280 290 300
EQHVQIDVDV SKPDLTAALR DVRQQYESVA AKNLQEAEEW YKSKFADLSE
310 320 330 340 350
AANRNNDALR QAKQESTEYR RQVQSLTCEV DALKGTNESL ERQMREMEEN
360 370 380 390 400
FAVEAANYQD TIGRLQDEIQ NMKEEMARHL REYQDLLNVK MALDIEIATY
410 420 430 440 450
RKLLEGEESR ISLPLPNFSS LNLRETNLDS LPLVDTHSKR TLLIKTVETR
460
DGQVINETSQ HHDDLE

Figure S6. Vimentin amino acid sequence. Arginine (yellow) and lysine (light blue) residues are highlighted.
